# Text mining for identifying the nature of online questions about non-suicidal self-injury

**DOI:** 10.1186/s12889-022-13480-7

**Published:** 2022-05-25

**Authors:** Myo-Sung Kim, Jungok Yu

**Affiliations:** 1 College of Nursing, Healthcare Sciences & Human Ecology, Dong-Eui University, Busan, South Korea; 2 College of Nursing, Dong-A University, Busan, South Korea

**Keywords:** Non-suicidal self injury, Internet, Data mining

## Abstract

**Objective:**

The internet provides convenient access to information about non-suicidal self-injury (NSSI) owing to its accessibility and anonymity. This study aimed to explore the distribution of topics regarding NSSI posted on the internet and yearly trends in the derived topics using text mining.

**Methods:**

We searched for the keyword “non-suicidal self-injury” (Ja-Hae in Korean) in the Naver Q&A using the statistical package R. We analyzed 7893 NSSI-related questions posted between 2009 and 2018. Text mining was performed using latent Dirichlet allocation (LDA) on the dataset to determine associations between phrases and thus identify common themes in posts about NSSI.

**Results:**

In the LDA, we selected the following 10 most common topics: anger, family troubles, collecting information on NSSI, stress, concerns regarding NSSI scarring, ways to help a non-suicidal self-injurious friend, depression, medical advice, ways to perform or stop NSSI, and prejudices and thoughts regarding non-suicidal self-injurious people.

**Conclusions:**

This study provides valuable information on the nature of NSSI questions posted online. In future research, developing websites that provide NSSI information and support or guidance on effectively communicating with NSSI is necessary.

## Introduction

Non-suicidal self-injury (NSSI) is a deliberate and consistent act of injuring one’s body without suicidal intent [1]. NSSI conflicts with the fundamental human behavior of avoiding pain and pursuing pleasure. In adults, the NSSI experience rate accounts for 4–23% but is considerably higher among adolescents, reaching 7.5–46.5% [2]. In a recent study, NSSI occurred in 8.8–22.8% of middle and high school Korean students [3]. Thus, NSSI among adolescents has elicited increased attention and concern worldwide.

NSSI is most commonly executed by burning, scratching, hitting parts of the body, interfering with wound healing, or skin-cutting with a knife [3, 4]. NSSI is likely to cause bodily damage and predicts suicidal behavior [5, 6]. Joiner [7] regarded NSSI as one of the many behaviors that can indirectly increase suicidal behaviors through acquired capability for suicide. Individuals with NSSI were significantly more likely to report higher suicidal ideation and suicide attempts than those who did not engage in NSSI [8]. Unfortunately, medical treatment is not provided in many cases because NSSI is generally performed secretly and personally due to social stigma and prejudice. Hence, it requires a multidisciplinary approach to understand the characteristics and needs of individuals who engage in NSSI.

The internet is a primary resource for information, especially finding or discussing sensitive or controversial topics. Recently, various NSSI photos and communities have appeared on the internet [9]. According to a systematic review study, individuals who engaged in NSSI asked their friends on social networking platforms for help instead of seeking professional assistance such as medical help [10]. Despite the need to examine the discussion about NSSI on the internet, studies regarding NSSI in Korea remain poorly investigated and are insufficient compared with social needs [3].

Studies on the increasing incidence of NSSI in adolescents have been actively conducted to discuss and analyze NSSI on social media and online. Such studies have investigated online questions about NSSI [11], responses to NSSI photos on the internet, and the positive or negative effects of online NSSI on participants [12]. However, most of these previous studies were limited to approximately 100 data posted within a short time and in the context of developed Western countries.

Text mining can identify massive text content on the internet appropriately. Text mining unearths hidden knowledge by finding patterns in a massive unstructured text [13]. Recently, a study demonstrated the usefulness of the text mining technique in identifying self-injuries on the internet by showing considerable agreement between topics derived by human beings and topics derived by topic modeling [14]. Using text mining, we can examine the information needs of NSSIs by analyzing NSSI in internet forums. The extracted concepts and meanings can be used as basic data to add to the understanding of how NSSI presents globally and to develop programs for non-suicidal self-injurious individuals.

This study aimed to derive topics of questions about NSSI by exploring Internet Q&A data related to NSSI posted on Naver Knowledge iN from 2009 to 2018 using the topic clustering technique of text mining. Our study has two specific objectives: to identify the distribution of NSSI question topics posted on the internet and determine the yearly trends of the derived topics.

## Methods

### Subjects and data

We used posts on the “Knowledge iN” of Naver, which is currently the leading search engine of South Korea, to extract NSSI information. “Knowledge iN” is a Naver knowledge search service and the first among domestic portal sites that opened in October 2002. As the Korean name implies, knowledge iN refers to human beings (iN). It is a community where people exchange their thoughts; anonymous users post questions to other users. Most of the respondents are the general public instead of experts in this peer-based Q&A service. Still, experts provide answers for some topics and areas where the service provider cooperates with professional organizations and individuals [15]. Anyone can access the contents without the need to sign in, but to write or answer a question, users must log in to a Naver account with the assurance of anonymity.

Since data from 2002 to 2008 is small amount of posts with 194 cases in total, it is not appropriate to identify topics and changes by year. Data from posts on Naver Knowledge iN (https://kin.naver.com) were collected from January 1, 2009, to December 31, 2018. The posts contained the term “non-suicidal self-injury (Ja-Hae in Korean).” In academic terms, Ja-Hae includes suicide and self-injury without suicidal intentions. However, in Korea, Ja-Hae is expressed as having no intention of suicide. So the general public and the media use Ja-Hae to denote harm to one’s body without suicidal intent and Ja-Sal to denote self-injury intended to commit suicide. Usually, creators express suicide a lot with the initial consonants letter of ‘Ja-Sal’ when they want to use it on Naver.

### Data collection

**Fig. 1:**
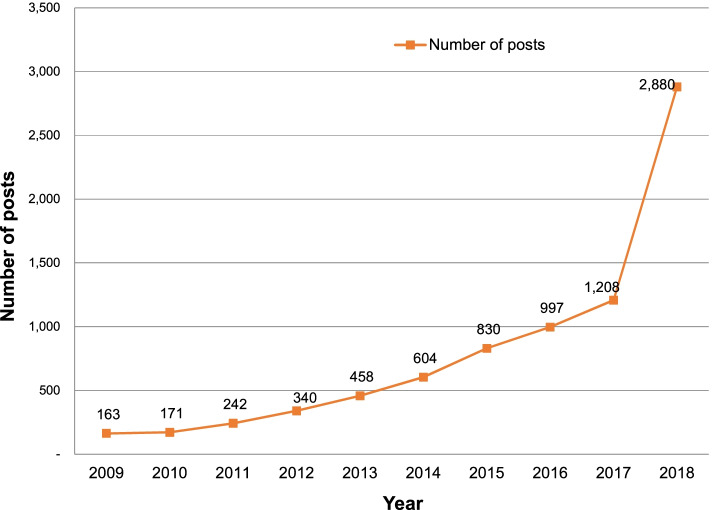
Number of posts included in the analysis by year

These data were collected using the query, “non-suicidal self-injury” (Ja-Hae in Korean). Naver designated specific self-injury behaviors, such as “wrist cutting,” as harmful keywords. Terms such as burning, scratching, and hitting were inappropriate because they were not self-injury-focused. The searched data were gathered using the program R’s httr, rvest, and stringr packages. The URL of the page searched with Ja-Hae was called in program R and saved as HTML. Among the data contained in the HTML, the text of the node with the title, content, and date of the post was extracted. The post’s collected title, content, and date were stored in CSV format. Naver only displays up to 1000 posts at a time when searching for data; therefore, data extraction was performed several times by dividing the period. We gathered 9013 posts that included Ja-Hae in their titles. However, 267 were redundant posts, and 177 were inappropriate for analysis because they only contained a title and no content. Posts with different URLs but the same title and content were considered redundant and identified using duplicate functions. Additionally, 676 posts were unrelated to non-suicidal self-injury (self-injury of animals, such as parrots and hamsters, and inclusion of Ja-Hae in other terms caused by spacing errors, such as “Chinese character interpretation [Han-Ja-Hae-Seok in Korean]”). Ultimately, 7893 posts were analyzed. Figure 1 shows the annual distribution of the data.

### Ethical considerations

This study examined text data from an internet community board on Naver (https://kin.naver.com). The board was anonymously operated, and the scope of the data collected for the study included titles and contents of posts, which did not contain personal information, such as the questioner’s username. All methods were performed following relevant guidelines and regulations [16, 17].

### Data analysis

#### Text preprocessing and word extraction

**Fig. 2:**
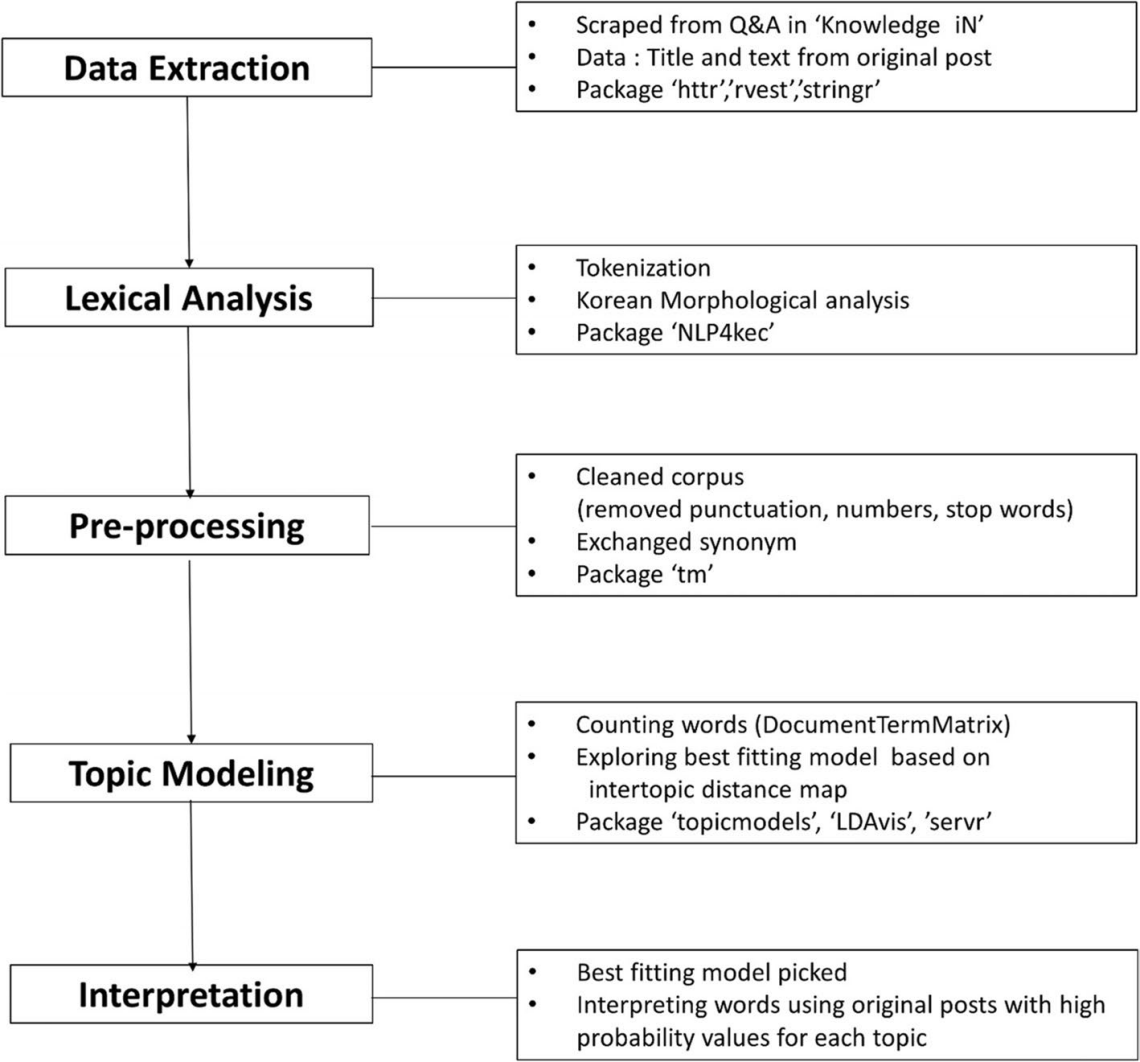
Flowchart of data processing

The collected data were preprocessed and analyzed using R version 3.4.2. For data preprocessing (Fig. 2), we converted the input character sequences into morpheme sequences through morphological analysis. For the morphological analysis, we utilized the NLP4kec package [18] to assist an open-source Korean morpheme analyzer called Eunjeon for natural language processing.

The data were preprocessed using the tm package in R. After converting the analyzed file into a corpus, we removed punctuation marks, special symbols, and numbers. Stop words are not useful for analysis and are typically extremely frequently used words. Some meaningless words (e.g., do, be, and become), dependent nouns (e.g., thing, place, spot, and as), pronouns (e.g., this, I, me, and my), and conjunctive adverbs were treated as stop words [19] and were thereby removed. Synonyms such as “homeroom teacher,” “tchr (ssam in Korean),” and “teacher” were unified into one word, that is, “teacher.” teacher.’

After the preprocessing step, we created a document term matrix (DTM) representing the frequency of terms across each document in the corpus. DTM presents documents and terms in rows and columns, respectively [20]. From the 7893 posts, 11,188 words were extracted. Sparse terms that did not appear in 98.0% of documents or more were removed, resulting in a DTM containing 245 of the most frequently used terms from the corpus.

#### Topic analysis based on latent Dirichlet allocation

To identify the distribution of NSSI information on the Internet forum, we analyzed topics using LDA [21], the most widely used topic modeling method. Hidden topics in the text were objectively identified using LDA topic modeling according to probability. According to the LDA algorithm, a single topic can be described as a multinomial distribution of words, whereas a single document can be described as a multinomial distribution of the latent topics. The model uses the observed documents and words to infer the hidden topic structure, creating per-document topic distributions P (topic|document) and per-topic word distributions P (word|topic). The probability of topics and terms was calculated by repeated sampling of the distribution and prior probability of the actual text data based on Bayesian inference [22]. We used the topicmodels, LDAvis and servr packages of R for LDA analysis.

The number of topics must be determined before topic extraction using LDA. Clear interpretations may not be achieved despite the proposal of a perplexity-based method. Considering that the researcher estimates the number of topics, justification logic must apply to the adequacy of the number of topics [21]. Accordingly, the similarity among topics was visually verified by generating an Intertopic Distance Map (IDM) via a multidimensional scale while varying the number of topics from 7-15. The distance between the bubbles shows the similarity between topics and identifies the degree of relevance between them. The IDM results revealed that topic redundancy was low for 10 topics. Subsequently, these 10 topics were chosen, as each of them could be interpreted independently. LDA outputs include the most probable words of each topic, topic probability of each document, and topic number with the highest probability value in each document. Consequently, 15 key terms were selected to represent each topic. The topic probability and topic number of each document were considered as outputs. Each topic was named according to the original questions with high topic probability and the correlation of terms extracted for each topic.

#### Yearly changes in topics

NSSI-related posts have increased rapidly in the past few years. In this study, the ratio of each topic to the total number of annual posts was calculated as a percentage from 2009 to 2018.

## Results

### Analysis of NSSI contents through topic analysis

Table 1 summarizes the topic analysis results for NSSI-related content, and 10 topics were derived. Each topic was named according to its corresponding key term, and the original documents were classified according to the topic name. The topics were called T1 (anger), T2 (family troubles), T3 (collecting information on NSSI), T4 (stress), T5 (concerns regarding NSSI scarring), T6 (how to help a self-injurious friend), T7 (depression), T8 (medical advice), T9 (how to perform or stop NSSI), and T10 (prejudices and thoughts regarding persons who engage in NSSI).

**Table 1:** The most probable words and quoted example in the topics of latent Dirichlet allocation

Topic number	Label	Words
T1	anger	anger, parent, hit, head, body, irritation, hand, suppress, punch, behavior, strike, strong, resolve, psychopathy, neck
	*When I’m angry, I slap myself very hard and punch myself in the head. If I express anger, my parents will scold me. Hitting myself seems to harm no one, so I keep doing it. Should I get counseling?* (2505)	
T2	family troubles	mother, father, eat, home, be hit, cry, swearing, fight, younger brother/sister, go out, alcohol, family, older sister, money, buy
	*I think the reason I first started hurting myself was because of my mother, who is addicted to gambling, and my father, who is usually caring but used to wield violence whenever he drank. When I entered middle school, my mom gambled away the money I needed to buy my school uniform. My home was a mess that day. I wondered why I was born home like this. (2194)*	
T3	collecting information on NSSI	cut, knife, blood, wrist, dorsum of hand, bleed, nail, tear, deep, finger, mechanical pencil, discharge, hand, first time, swell
	*I love the barcode of the red lines listed under the wrist. I feel the scars on the upper arm are beautiful. It’s become a daily life now. Why am I doing this? It’s not that I don’t have hobbies. However, they can’t replace.* (834)	
T4	stress	stress, severe, feeling, problem, study, terrible, relieve, sensation, family, girl student, big, anxiety, feel heavy, solution, word
	*Whenever I’m stressed, I hurt myself. Especially when it’s like a stressful exam period. I know I shouldn’t harm myself, but I relieve my stress so well that I can’t stop it.* (777)	
T5	concerns regarding NSSI scarring	scar, wound, remain, cover, arise, part, photograph, apply, bandaid, regret, heal, remove, ointment, stick, disappear
	*I’m so concerned about the scar. Even in winter, I wear short-sleeved clothes, but now I have no choice. I apply ointment regularly, but it doesn’t disappear immediately. I’m so scared that I’ll get caught.* (1549)	
T6	how to help a self-injurious friend	friend, school, counsel, teacher, talk, attend, time, cry, boyfriend, console, close, laugh, meet, live, play
	*I saw my friend’s scar. After that, I went around looking at all my friends’ wrists, and there was another friend who was hurting herself. I want to comfort and help my friends, but my friends feel burdened by me. Can I tell teacher or counselor? What should I do?* (2278)	
T7	depression	hard, die, depression, help, suicide, mind, impulse, okay, sad, try, emotion, do not know, mental hospital, comfort, mentality
	*I was very negative and depressed for a long time. But I’ve never felt this big before. I keep hurting myself because I feel like I’m going to die. I want to kill myself, but I’m scared. I hope someone tells me to die. Please... Then there’s a reason to die.* (862)	
T8	medical advice	arm, method, appear, hospital, wrist, mental, scratch, treatment, stop, medicine, condition, mark, addiction, wear, left
	*There are about four marks on the left arm and wrist, but I want to treat them neatly. Which hospital can I treat it at? I’m studying a major in a field that shouldn’t have scars, so I need treatment.* (2259)	
T9	how to perform or stop NSSI	pain, do not know, begin, inform, first time, rise up, woman, reveal, stop, method, treat, interest, long, ask, concern
	*Is there any part where you can reduce pain and see wounds and blood when you hurt yourself?* (3648*) It hasn’t been long since I started, but I end up doing it even if I don’t want. Can you tell me how to stop?* (3460)	
T10	prejudices and thought about self-injurious people	thought, people, live, hate, inferior, feel, reason, mad, alone, bad, do not know, mind, near, suffering, love
	*Self-harm is bad, but people who harm themselves are not bad. It’s pitiful. It’s not dirty. I’m not crazy.* (1513)	

On analyzing the content of each topic, T1 had questions on whether self-injury as a response to anger was abnormal and whether counseling was required. T2 had queries about how to respond to self-injury or injury by family members caused by parental discordance or unstable family situations. T3 had posts where individuals questioned whether their specific behaviors, like scratching, tearing, or hitting a body part, corresponded to self-injury and why they behaved in these ways. T4 included posts on how one could stop self-injuring under stress. T5 had questions regarding the concealment of scars created by NSSI and scar removal solutions, such as the use of an ointment. T6 included questions about helping a friend prone to NSSI. T7 had complaints about distressing situations that caused depression, sorrow, self-injurious thoughts, and suicidal ideation. T8 dealt with the treatment of NSSI through hospitalization and the treatment of traces of self-injury in the past. T9 had questions from people who had not yet experienced NSSI; they asked whether self-injury was painful and how they could self-injure without pain. In addition, this topic included questions about ways to stop NSSI. They were concerned that they might progress into more severe self-injury and could not feel any pain during NSSI. Finally, T10 had queries on why society derided NSSI or how others perceived people engaging in NSSI. Some of them asked how one could cope with the situation if they did not dare to tell others that they unavoidably engaged in NSSI.

Of the 7895 documents, 18.0% were concerned about NSSI scarring (topic 5), followed by 14.7%, which were questions about whether one’s behavior was NSSI (topic 3) (Table 2).

**Table 2:** Topic allocation rate by year

topic	T1	T2	T3	T4	T5	T6	T7	T8	T9	T10	Sum (n)
year	percentage										
2009	8.6	17.2	19.6	5.5	6.1	9.8	4.3	9.8	5.5	13.5	163
2010	13.5	18.7	15.8	5.8	6.4	6.4	6.4	4.7	5.8	16.4	171
2011	7.9	15.7	17.4	7.0	11.2	11.6	5.4	7.9	5.4	10.7	242
2012	11.5	12.4	19.7	6.8	10.3	9.7	5.3	5.9	5.9	12.6	340
2013	11.1	7.0	19.7	8.7	15.5	6.8	7.0	9.2	5.0	10.0	458
2014	11.8	9.4	14.7	7.9	13.6	8.1	7.0	7.3	8.9	11.3	604
2015	11.3	8.9	15.2	8.9	15.7	7.2	7.3	6.3	7.7	11.4	830
2016	9.7	6.9	16.1	9.0	16.3	9.2	5.5	8.9	7.7	10.4	997
2017	9.2	9.1	16.1	10.2	16.8	9.3	8.0	6.1	6.5	8.7	1208
2018	5.7	6.0	11.5	6.6	24.0	11.7	11.0	7.6	7.3	8.8	2880
Sum n (%)	682 (8.6)	654 (8.3)	1159 (14.7)	623 (7.9)	1422 (18.0)	768 (9.7)	654 (8.3)	582 (7.4)	558 (7.1)	791 (10.0)	7893

### Trend of yearly changes in topics

**Fig. 3:**
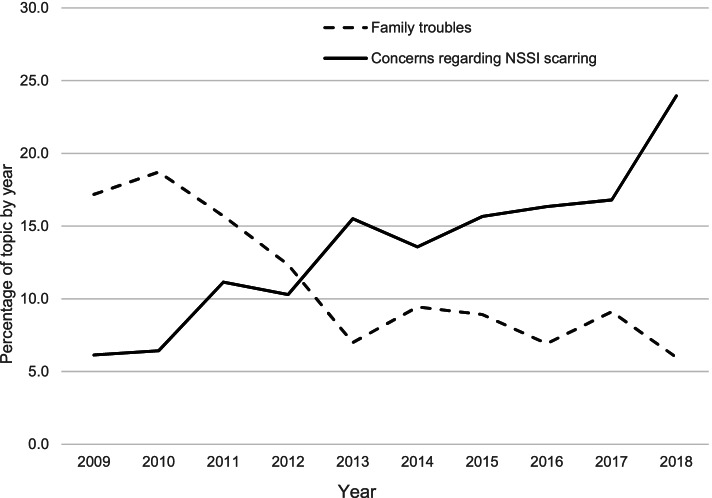
Dynamics of the most and least talked-about topics from 2009 to 2018

Table 2 lists the topic numbers derived in each year. Given that posts about NSSI continued to increase, examining yearly changes simply by using frequency was difficult. When converted into a percentage, the proportion of posts about topic 3 (collecting information on self-injury) tended to decrease by year (from 19.6 to 11.5%) but showed a high rate of topics every year. The proportion of posts about topic 5 (NSSI scarring) increased rapidly (from 6.1 to 24.0%). Conversely, the proportion of posts about topic 2 (NSSI caused by family troubles) decreased (from 17.2 to 6.0%) (Fig. 3). In addition, topic 10 (prejudices and thoughts regarding persons who engage in NSSI) tended to decrease compared to before (from 13.5 to 8.8%), but at a slower rate than topic 2.

## Discussion

This study analyzed 7893 Naver Knowledge iN posts through topic modeling to understand online posts regarding NSSI. Overall, posts on NSSI increased continuously from 2009 to 2018. Moreover, we identified 10 topics and keywords for each topic.

Recent figures have shown a dramatic increase in the number of people who self-injure in South Korea. In 2018, the number of NSSI counseling sessions at youth counseling and welfare centers more than tripled compared to that in 2017 [23]. In addition, as shown in Fig. 1, the data collected in this study tripled in 2018 compared to 2017. The cause of the sudden increase in the number of posts in 2018 is not clear. Naver Knowledge iN represents a general question and answer community that permits users to provide and receive information in Korea. Although its primary goal is to give and seek information, people engaging in NSSI seem to seek information and emotional support for NSSI. People worldwide have used the internet as a means of communication, given that it offers a favorable environment for users to discuss sensitive or unexposed health topics [24]. This finding suggests the need for consultative interventions in which self-injured people can receive help online.

Of the 10 topics derived from this study, the topic of “concerns regarding NSSI scarring” (T5, 18.0%) had the greatest number of questions, and the number rapidly increased yearly. Similar to the results of a previous study [25] in which many individuals engaging in NSSI were conscious of their wounds or scars and made desperate efforts to hide wounds from others, our study found many questions about the method of hiding scars. Such questions included, “Does NSSI leave scars?” and “How can I hide scars from the NSSI?” However, questions about the treatment of scars created in the past, hospitals, departments that can treat scars, treatment methods, and the treatment of complications were classified as another topic named “medical advice” (T8, 7.4%). This topic included questions regarding hospital treatment for NSSI. Given that Naver Knowledge iN represents a general Q&A community, informational needs regarding NSSI, such as concerns regarding scarring and medical advice, are the most commonly asked questions. Consistent with previous online forum content [11], the current study demonstrated that scar concealment is common. Many individuals hide scars and treat them secretly in private because of social stigma [26]. However, considering that NSSI can induce serious safety issues by causing infection or secondary disease of wounded parts, in addition to scars formed on the skin, individuals need to understand the importance of adequate wound treatment and inform them about coping methods. In addition, a previous study [27] indicated that the degree of scar concealment is associated with more significant experiences of higher levels of anxiety and depressive symptomatology and higher severity of NSSI urges. The increase in posts on NSSI scarring concerns means that a more in-depth understanding of the scar concealment practices of individuals engaging in NSSI is needed.

The second-largest number of questions collected in this study were about “collecting information on NSSI” (T3, 14.7%). This topic described NSSI in detail and included questions on whether a specific behavior corresponds to NSSI and why one would behave that way. In particular, the term “first time” was ranked top, indicating that people who started NSSI use the internet to gain information. Therefore, efforts are needed to provide NSSI information and support on the internet. Additionally, the accuracy of the information on websites should be monitored and evaluated to protect individuals against unhelpful responses [10].

Another topic considered was “prejudices and thoughts about self-injurious people” (T10, 10.0%). Individuals who shared their NSSI experiences wanted acceptance and verification more often than those who did not disclose NSSI [11]. Individuals who engage in NSSI find it hard to reveal their condition to others for fear of stigmatization but want others to understand them because they cannot avoid NSSI at present. Instead of condemning the self-injurious behavior of individuals, we need to protect and support them within social and cultural contexts [28].

Furthermore, people who did not engage in NSSI asked questions about “how to help a self-injurious friend” (T6, 9.7%), signifying the importance of understanding as well as the demand of interested parties (e.g., family, friends, partners, and colleagues) who can seek answers to NSSI questions on the internet [9 Whitlock, Purington, and Gershkovich [29] reported that a person who witnesses NSSI or the scars of a friend might end up imitaion of NSSI when encountering difficult circumstances. NSSI prevention programs should be developed while considering the effects on self-injurious adolescents. Moreover, educating and utilizing peers who provide help to self-injurious friends is deemed beneficial [28].

We identified negative emotions such as anger (T1, 8.6%), depression (T7, 8.3%), and stress (T4, 7.9%) in the NSSI online posts. NSSI is a pathological response that functions as a coping mechanism to temporarily lessen intense feelings such as anxiety, depression, stress, emotional insensibility, sense of failure, and self-hatred [4, 28, 30]. NSSI stabilizes intense and unbearable emotions. It seems to control emotions when one tries to regain [31]. The topic of “depression” (T7) in this study included suicidal impulses. NSSI is highly correlated with suicide attempts, and suicide is strongly linked with depression [24, 25, 28]. A meta-analysis of the prevalence of NSSI functions in the community and clinical samples [30] showed that intrapersonal functions (e.g., concerning emotion regulation) were most reported by individuals who engaged in NSSI (63–78%), whereas interpersonal functions (e.g., expressing distress) were less common (33–56%). Several theoretical models for NSSI center on emotion regulation or the avoidance function of NSSI [30]. Although these models may fit better for individuals whose dominant motive driving NSSI is regulating difficult emotions, they may fit poorly for someone whose primary function concerns self-punishment or distress communication.

The other topic was “how to perform or stop NSSI” (T9, 7.1%). Individuals interested in NSSI were asked whether NSSI is painful and how one can self-injure without pain; thus, internet activities can be used as a means of sharing methods of NSSI. This topic also included questions about ways to stop self-injury; they were concerned that they might progress into a more severe self-injury and would not feel any pain during NSSI. Hooley et al. [32] found that individuals who engage in NSSI demonstrated greater pain tolerance and pain thresholds in lab-based tasks than non-injuring control groups, suggesting that NSSI, in particular, maybe one mechanism through which individuals become desensitized to pain [8, 32]. We predict that there will be a stronger association between NSSI and acquired capability among individuals engaging in severe or repeated NSSI. According to acquired capability which is a construct in the Interpersonal Theory of Suicide by Joiner, once an individual attains the acquired capacity for suicide through desensitization or habituation to fear and pain, it is likely that an individual will be at increased risk for suicide [7]. However, according to a study analyzing Instagram posts, only 1% of all posts conveyed information or messages about the need to help reduce NSSI [24]. The Disaster and Trauma Information Center provides fragmented information on NSSI in Korea, but Korean websites lack information and public services related to NSSI. According to Frost and Casey [33], various channels, including online support for NSSI, are necessary to assist adolescents and young adults who cannot seek help through other means. Individuals use diverse types of online support and differ in what support they seek. An exhaustive understanding of the characteristics and scope of online activities of self-injurious individuals can help promulgate self-injury interventions [11]. Internet posts have the positive aspects of expressing and communicating negative emotions, but there are also negative aspects associated with sharing NSSI experiences and finding ways to harm themselves. Because NSSI material in these online communities may normalize and reinforce NSSI, monitoring and intervention may be necessary to ensure that people who do and do not harm themselves do not receive inappropriate and unhelpful feedback on the internet. Mental health professionals need to participate in question-and-answer communities for appropriate online interventions provided by groups of experts to meet the needs of those who engage in NSSI.

NSSI caused by the discordance between parents and unstable family situations was categorized as “family troubles” (T2, 8.3%), and the number of questions regarding it decreased yearly. Emotional deprivation of parents, dysfunctional family, and an unstable nurturing environment in childhood are risk factors for NSSI [28]; hence, family factors must be included when counseling self-injurious individuals. Considering that family support has the most significant effect on the beginning, maintenance, and cessation of NSSI [34], investigating the relationship between a self-injurious child and parents would help prevent NSSI.

## Limitations

This study extracted data using the keyword “non-suicidal self-injury (Ja-Hae in Korean)” when identifying types of questions related to NSSI on Naver. Although the extracted data contained the interests and voices of people who asked self-injury-related questions, this study classified data based on frequently used terms and did not conduct an in-depth analysis of the massive amount of data collected. Considering that our data were obtained from a Korea-only website, the generalizability of this study is limited. Given the anonymous nature of Naver, personal demographic information of the questioner, such as age and gender, was unavailable. Therefore, examining potential sex and age differences regarding posts on NSSI on Naver was not possible.

## Conclusions

The current study demonstrated that the number of NSSI-related internet posts increased dramatically over the years, consistent with the number of face-to-face NSSI-related consultations [23]. This study identified the general question-and-answer internet communities such as Naver is the crowdsourcing platform for people to obtain information and support regarding NSSI across a range of different aspects of the behavior. Additional research is needed to investigate different online platforms such as Twitter and Instagram, as this can inform mental health professionals meeting with individuals who engage in self-injurious behaviors about the nature of online activity regarding NSSI.

## Data Availability

Raw data can be extracted through setting period and keyword on Naver (https://kin.naver.com/qna/list.naver). The datasets used and/or analysed during the current study are available from the corresponding author on reasonable request.
